# Real-Time Rotational Activity Detection in Atrial Fibrillation

**DOI:** 10.3389/fphys.2018.00208

**Published:** 2018-03-13

**Authors:** Gonzalo R. Ríos-Muñoz, Ángel Arenal, Antonio Artés-Rodríguez

**Affiliations:** ^1^Signal Theory and Communications Department, Universidad Carlos III de Madrid, Madrid, Spain; ^2^Gregorio Marañón Health Research Institute, Madrid, Spain; ^3^Department of Cardiology, Hospital General Universitario Gregorio Marañón, Madrid, Spain

**Keywords:** atrial fibrillation, multi-electrode catheter, signal processing, real-time, rotors, rotational activity

## Abstract

Rotational activations, or spiral waves, are one of the proposed mechanisms for atrial fibrillation (AF) maintenance. We present a system for assessing the presence of rotational activity from intracardiac electrograms (EGMs). Our system is able to operate in real-time with multi-electrode catheters of different topologies in contact with the atrial wall, and it is based on new local activation time (LAT) estimation and rotational activity detection methods. The EGM LAT estimation method is based on the identification of the highest sustained negative slope of unipolar signals. The method is implemented as a linear filter whose output is interpolated on a regular grid to match any catheter topology. Its operation is illustrated on selected signals and compared to the classical Hilbert-Transform-based phase analysis. After the estimation of the LAT on the regular grid, the detection of rotational activity in the atrium is done by a novel method based on the optical flow of the wavefront dynamics, and a rotation pattern match. The methods have been validated using *in silico* and real AF signals.

## 1. Introduction

Atrial fibrillation (AF) is one of the most frequent sustained arrhythmias in clinical practice (Kirchhof et al., 2016), and is associated to increased morbidity (heart failure, ictus) (Wakili et al., 2011). The underlying mechanisms initiating and sustaining AF are still under debate, and this situation prevents electrophysiologists to cure the arrhythmia using catheter ablation and/or antiarrhythmic drugs.

The traditional theory for AF maintenance relies on multiple wavelets propagating at random in the atrial tissue (Moe, 1962; Allessie et al., 1985). The wavelet hypothesis states that a minimum number of simultaneous wavelets would perpetuate AF. However, its maintenance possibly involves some form of reentry circuit caused by wavebreaks (Shiroshita-Takeshita et al., 2005).

Alternatively, spatiotemporal stable sources (rotors) were proposed as the maintenance mechanism of AF (Jalife et al., 2002). In *in silico* simulations and preclinical experiments rotors are formed and maintained when a wavefront interacts with obstacles, scars, or heterogeneous tissues with anisotropic conduction. The temporal stability characteristic of a rotor and its spiral wave pattern, compared to the multiple wavelet irregular propagation, facilitates the development of new algorithms that could detect and confirm the role of rotors as AF drivers. Unfortunately, in the clinical practice the role of rotors as AF drivers is still controversial, with no confirmation nor acceptance of the rotor paradigm (Allessie and de Groot, 2014a,b; Narayan and Jalife, 2014).

In recent years new solutions have been proposed to study the existence of rotors and their relationship with AF termination. These works detect and characterize drivers using imaging of complex activation patterns in real-time. Specific examples are the invasive systems RhythmView (Topera) (Narayan et al., 2012), CartoFinder (Biosense Webster) (Daoud et al., 2017), AcQMap (Acutus Medical) (Grace et al., 2017; Martin et al., 2017), and non-invasive CardioInsight ECVUE (Medtronic) (Yamashita et al., 2015).

The RhythmView system, one of the leading technologies in clinical practice, was developed as part of the Focal Input and Rotor Modulation (FIRM) method (Narayan et al., 2012). For the detection of rotors, the method requires two basket catheters deployed in both atria, exporting the data to perform offline signal processing, and one trained operator to determine the presence of the rotors. The method is based on the detection of electrode local activation to construct isochronal maps. Then, it performs phase analysis by directly applying the Hilbert transform to unipolar EGMs to detect phase singularity points where the tip of the rotor spins (Gray et al., 1998).

Despite of its promising preliminary results, the above described method presents several limitations: The phase mapping correlates poorly with temporal activation maps (Vijayakumar et al., 2016). The method needs two basket catheters, which is intrusive for the patient. The catheter topology presents no efficient deployment and electrode contact (Laughner et al., 2016). The low spatial resolution mapping of the atria, is prone to false detections (Roney et al., 2017). The solution requires signal exportation plus post-processing, which extends the duration of the clinical procedure and prevents reproducibility of results (Benharash et al., 2015; Buch et al., 2016).

Similar to RhythmView, the novel CartoFinder software was developed to detect and characterize drivers in AF (Daoud et al., 2017), with recent studies confirming rotational activations (Calvo et al., 2017). This solution also employs a basket catheter to acquire the signals and identify rotational repetitive activation patterns (RAPs). The main difference with respect RhythmView resides in the identification of the unipolar activation in a determined time window defined by the bipolar information of the electrode pairs. The detection of RAPs is based upon visual inspection. The compatibility with the Carto 3D electroanatomical system allows CartoFinder to project the activations onto the electroanatomical 3D map, instead of unfolding the atrium into a 2D grid for visualization. However, as it requires the use of basket catheters, CartoFinder presents the same limitations in terms of electrode deployment and atrial contact as RhythmView.

The AcQMap system employs a basket catheter with 48 electrodes and 48 ultrasound transducers to perform non-contact 3D electroanatomical reconstruction and signal acquisition. The ultrasound technology generates maps faster than by using traditional mapping catheters. The electrical activations are calculated with algorithms applied to intracardiac voltage signals and represented as unipolar voltage and Dipole Density™ maps. This system has the advantage of embedding the generated activation maps in the 3D anatomical model for visualization. Nevertheless, the non-contact feature is sensitive to the distance of the electrode to the atrial wall and catheter positioning.

Sharing the same goal, non-invasive methods aim to characterize AF prior to the surgical procedure. One example is the CardioInsight ECVUE, based on a multi-electrode vest recording body surface electrocardiograms (ECGs) which are combined with CT scan data. While the system is able to display 3D cardiac epicardial activation maps, it has potential limitations compared to invasive approaches which map endocardial tissue. The assessment of drivers is limited in some particular regions like the septal area, and some tissues between the body surface and the epicardium may affect the signals. Finally, the signal to noise ratio of the system limits its accuracy in the detection for short and small amplitude drivers, and it may not correlate with reentries identified by other systems, although important advances focusing on demonstating this correspondance have been done (Rodrigo et al., 2017).

In this work, we present a new automatic rotational activity detection method that operates in near real-time with atrial contact. In contrast to other invasive mapping methods, our system can be easily configured for different multi-electrode topologies (e.g., circular, spiral, pentameric). This avoids contact problems and allows electrophysiologists to study if reentries can be explained by micro-rotors (which are too small to be mapped with other catheter models or missed by phase analysis approaches).

To our knowledge this is the first near real time rotational activity detection methodology providing multiple catheter topology compatibility. The proposed methods have been validated in simulated, experimental and clinical environments by means of direct comparison with other methods and error performance measurements.

## 2. Materials and methods

### 2.1. Objectives and validation

The objective of the system is to achieve rotational activation detection in real-time with a new methodology based on optical flow. For the validation of the methods we employed real AF signals acquired from patients with persistent AF and *in silico* simulations. The details on signal acquisition are included in the subsection Rotational Activation Detection System, and the *in silico* scenarios are presented in subsection Interpolation. Experienced electrophysiologists validated the outcomes of the methods and assessed the utility of the system, as shown in section 3 Results.

### 2.2. Rotational activation detection system

**Figure 1:**
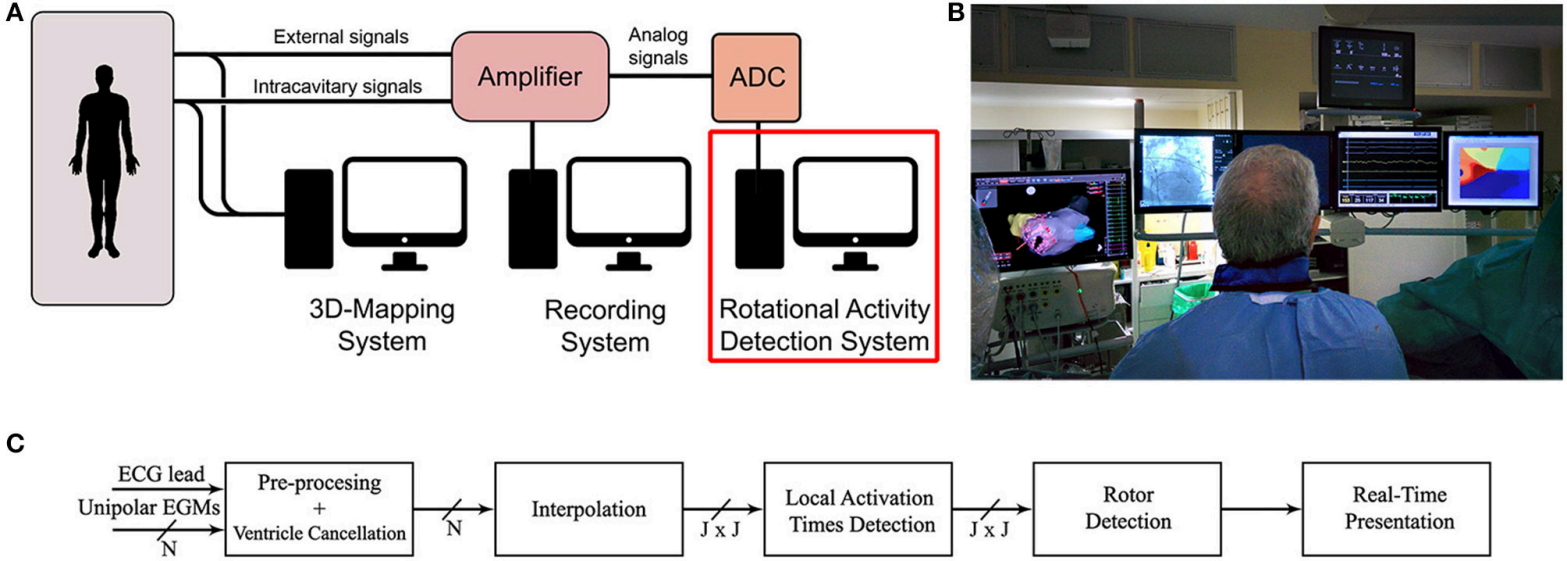
System implementation. **(A)** Common equipment in the electrophysiology laboratory. **(B)** Operating room monitors displaying the real-time rotational activity detection solution in the rightmost monitor. **(C)** Signal processing unit diagram implementing the rotational activity detection system. It receives as inputs N unipolar EGMs and one ECG lead, processes the signals and presents results in real-time.

The rotational activity detection system is based on an analog to digital converter (ADC), a processing unit and a monitor to display the results. The system can be easily integrated with other equipment commonly used in electrophysiology laboratories, as shown in Figures 1A,B. In this article we consider a pentametric shape catheter that consists of five branches with 4 electrodes, 20 in total, but other models can be used, i.e., circular, spiral, or basket.

The amplifier at the laboratory provides unipolar intracavitary EGMs from a multi-electrode catheter, and a reference external ECG. Although bipolar signals are preferred in clinical practice because of the far-field cancellation feature, they lack to provide precise local electrical activation information, as the electrical activation time instant cannot be accurately identified (Shenasa et al., 2009). For this reason unipolar configuration is preferred as the electrical activation timing is well defined by the point of maximum negative slope, and therefore activation maps can be built, on the expense of recording far-field ventricle activity, which can be later removed by using signal processing techniques. Other mapping solutions, i.e., RhythmView, CartoFinder, or AcQMap, also employ unipolar signals (Narayan et al., 2012; Daoud et al., 2017; Grace et al., 2017).

The sequence of operations to detect rotational activity from the acquired signals is shown in Figure 1C. We remove the baseline wandering present in unipolar signals and cancel the ventricle contribution to isolate the atrial activations. Then, we approximate the slope of the unipolar deflections related to the activation times of the atrium. By applying signal spatial interpolation on a regular bidimensional grid, we achieve independence from the catheter topology employed for mapping the atrium. Finally, we identify atrial activation times and perform the rotational activity detection. The results are presented in real-time in a monitor inside the operating room (Figure 1B).

### 2.3. Signal pre-processing and ventricle cancelation

**Figure 2:**
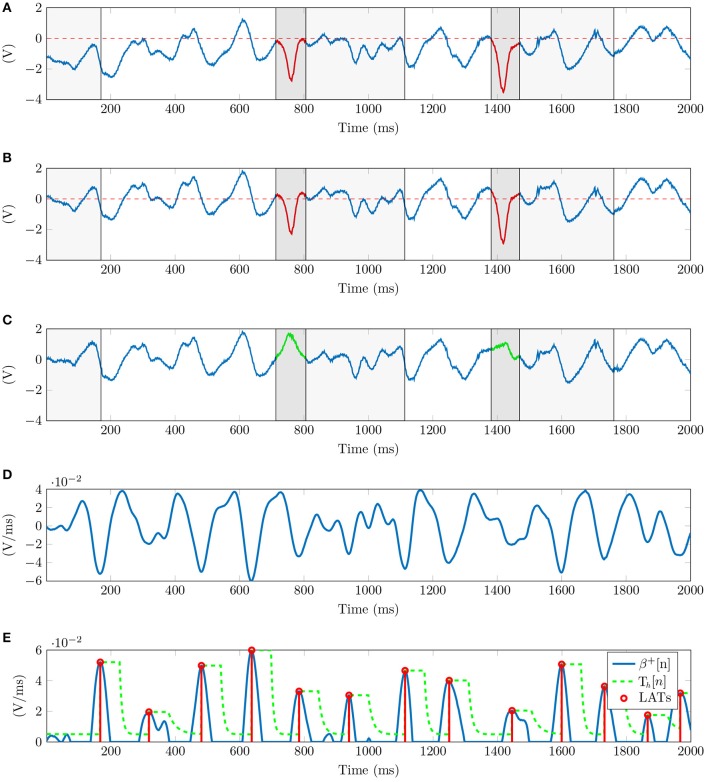
Signal processing for unipolar EGMs. **(A)** Raw unipolar EGM with ventricle contribution overlaying atrial activations delimited in dark and light gray for the QRS and ST intervals respectively. Level zero DC in dashed red shows the wandering DC offset affecting the signal. **(B)** Signal after baseline wandering correction. **(C)** Signal after ventricle cancellation, recovering occluded atrial activations shown in green. **(D)** Linear pattern approximation of the unipolar slope β[*n*] (*M* = 30 ms in the example). **(E)** Unipolar LATs in red detected from β^+^[*n*] in blue using an exponential decaying threshold, *T*_*h*_[*n*] in dashed green line.

Unipolar signals suffer from low frequency baseline drift caused by the respiratory movement of the patient (see Figure 2A). We estimate the DC signal component applying a causal median filter of 500 ms window length (de Chazal et al., 2003), and subtract it from the unipolar signal (Figure 2B).

Unipolar recordings are also affected by far-field signal contributions. The stronger ventricle signal overlays the atrial activity which has lower-amplitude, occluding atrial activations in the EGMs recordings (see Figure 2B). We cancel the ventricle contribution and recover hidden atrial activations calculating the ventricle unipolar pattern affecting each electrode signal, similarly to the average beat subtraction method described in the literature (Slocum et al., 1985), and current EP mapping solutions (Daoud et al., 2017). We calculate independent patterns for each channel as the electrodes record different atrial positions. To this end, the catheter is assumed to be stationary during the signal acquisition.

**Figure 3:**
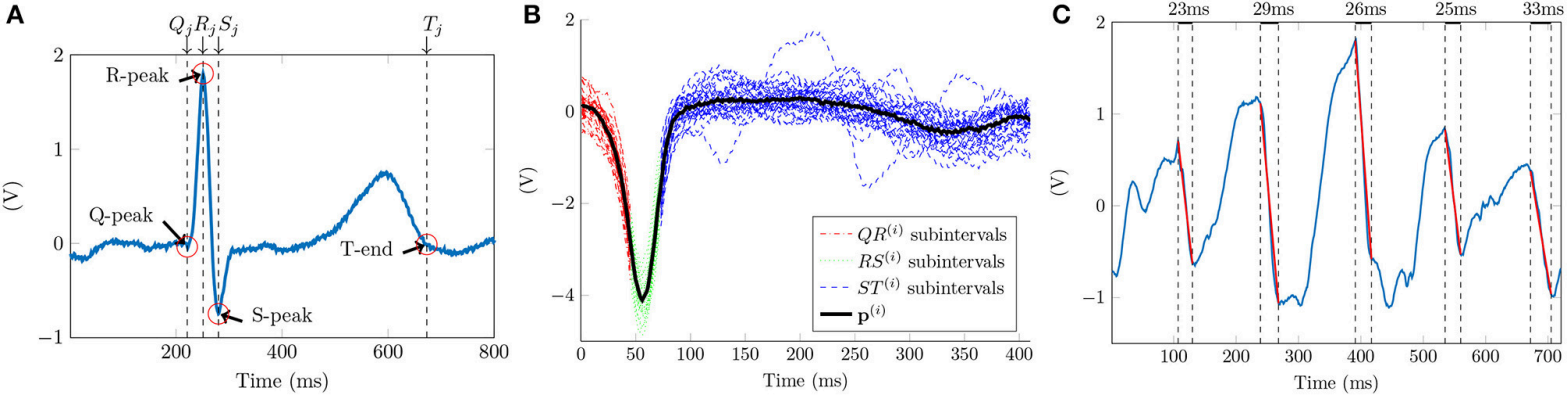
Ventricle cancellation in unipolar EGMs. **(A)** AF ECG with the reference points highlighted in red used for calculating the ventricle pattern. We can see the absence of a P wave, characteristic of AF since the atrium is not properly depolarized. **(B)** Averaged ventricle pattern in black calculated using 36 segments of unipolar channel i. Segments corresponding to the QR, RS, and ST subintervals are represented in red, green and blue respectively. **(C)** Unipolar recording after ventricle cancelation with depolarizations in red and their durations annotated between dashed lines in black.

A reference ECG signal identifies the scope of the ventricular contribution in the unipolar signals. The ventricle onset and offset are associated in the ECG to the Q-peak and to the T-wave end time instants respectively, as Figure 3A shows. The number of beats included for analysis varies depending on the heart rate of the patient and the duration of the acquisition. For this reason we set the minimum signal acquisition time to be at least 10 s, i.e., 10 beats for a 60 beats per minute rhythm. We guarantee at least 5 consecutive QRST complexes for a minimum heart rate of 30 bpm. Having 5 QRST complexes is enough to perform ventricle cancellation as demonstrated in other studies (Laughner et al., 2016). The 2nd Discrete Wavelet Transformation (DWT) scale of the ECG signal, using Daubechies DB4 wavelet, detects the R-peaks locations $R_{j}^{\left(i\right)}$ (Chui, 1992). Superscript *i* = 1, …, *N* corresponds to the *i-th* channel, and subscript *j* = 1, …, *J* refers to the *j-th* ventricle activation in the ECG signal containing a total of *J* activations. This scale corresponds to the 0−125 Hz frequency band of the signal for a sampling frequency *f*_*s*_ = 1 KHz. This frequency band covers the typical 0.05−100 Hz processing bandwidth for diagnostic ECG signals (Venkatachalam et al., 2011).

We threshold the signal and detect the R-peaks. A local search (±20 ms) corrects the $R_{j}^{\left(i\right)}$ time shifts when the DWT peak is converted back to the original scale. Once $R_{j}^{\left(i\right)}$ is found, a local search to the left of R-Peaks obtains the Q-Peaks in the ECG signal. The minimum in the $[R_{j}^{\left(i\right)}-50$ ms$R_{j}^{\left(i\right)}-10$ ms] interval corresponds to the $Q_{j}^{\left(i\right)}$ locations. Following the same searching procedure, the S-Peak is the minimum located within the $[R_{j}^{\left(i\right)}+5$ ms, $R_{j}^{\left(i\right)}+50$ ms] temporal window. An algorithm designed for positive T-waves detects the T-wave ends (Zhang et al., 2006). From all the external ECG leads the method needs one exhibiting T-wave concave morphology, i.e., lead I, V3, V4, or V5. The area under the curve is calculated using a 32 ms length overlapping sliding window with a one sample shift in an interval containing the T-wave. The time instant maximizing the area value gives the $T_{j}^{\left(i\right)}$ location.

Then, for each i-th channel we align all the unipolar segments in the range $\left[Q_{j}^{\left(i\right)},T_{j}^{\left(i\right)}\right]$ and obtain the ventricle pattern as the median of the channel segments (Figure 3B). The T-wave contribution to the unipolar signal is smaller than the QRS complex, for this reason we represent the QS and ST sub-intervals overlaying the atrial signal in dark and light gray respectively in Figures 2A–C. To cancel every ventricle contribution, the pattern and each segment are aligned before subtraction. The time shift is given by the maximum correlation time instant between the two signals. Finally, the pattern subtraction removes the ventricle contribution from the unipolar segment (Figure 2C).

### 2.4. Detection of local activation times

Depolarizations are characterized by an abrupt deflection of the action potential recorded by the catheter. Depending on different factors (i.e., conduction speed, atrium area, antiarrhythmic drugs) the downward slope duration of an atrial activation varies. Figure 3C shows this variation with falls lasting 23, 29, 26, 25, and 33 ms. We propose a new method to identify local activation times (LATs) in EGMs by searching a pattern exhibiting a linear deflection.

We approximate the EGM signal *x*[*n*] by the linear function in the interval defined by a 2*M* + 1 samples window centered at time instant *n*_0_, expressed as:

(1) $$\begin{array}{l} \hat{x}\left[n\right]=\beta \left[n_{0}\right]\left(n-n_{0}\right),\text{ for }n\in \left[n_{0}-M,n_{0}+M\right], \end{array}$$

where β[*n*_0_] represents the function slope value at time *n*_0_. We estimate β[*n*_0_] by minimizing the Mean Square Error (MSE) of the error function ζ[*n*_0_] defined as:

(2) $$\begin{array}{l} \zeta \left[n_{0}\right]=\overset{M}{\underset{n=-M}{\sum }}|x\left[n_{0}+n\right]-\hat{x}\left[n\right]{|}^{2}=\overset{M}{\underset{n=-M}{\sum }}|x\left[n_{0}+n\right]-\beta \left[n_{0}\right]\cdot n{|}^{2}. \end{array}$$

We include the ${\hat{\beta }}_{MSE}\left[n_{0}\right]$ derivation. We calculate the first derivative of ζ[*n*_0_] with respect to β[*n*_0_], and set it to zero:

(3) $$\begin{array}{l} \frac{\partial \zeta \left[n_{0}\right]}{\partial \beta [n_{0}]}=\frac{\partial }{\partial \beta [n_{0}]}\left\{\sum_{n=-M}^{M}|x[n_{0}+n]-\beta [n_{0}]\;\cdot \;n{|}^{2}\right\}\\ \;=\sum_{n=-M}^{M}\left\{-2n\;\cdot \;x[n_{0}+n]+2n^{2}\cdot \beta [n_{0}]\right\}=0.\; \end{array}$$

Solving for β[*n*_0_] in Equation (3), we obtain the value of β[*n*_0_] minimizing the MSE,

(4) $$\begin{array}{l} {\hat{\beta }}_{MSE}\left[n_{0}\right]=\frac{\overset{M}{\underset{n=-M}{\sum }}n\cdot x\left[n_{0}+n\right]}{\overset{M}{\underset{n=-M}{\sum }}n^{2}}. \end{array}$$

The denominator of Equation (4) is a constant that depends on the window length *M* which simplifies the expression to:

(5) $$\begin{array}{l} {\hat{\beta }}_{MSE}\left[n_{0}\right]=M\cdot \overset{M}{\underset{n=-M}{\sum }}n\cdot x\left[n_{0}+n\right], \end{array}$$

where $M={\left(\overset{M}{\underset{n=-M}{\sum }}n^{2}\right)}^{-1}$.

**Figure 4:**
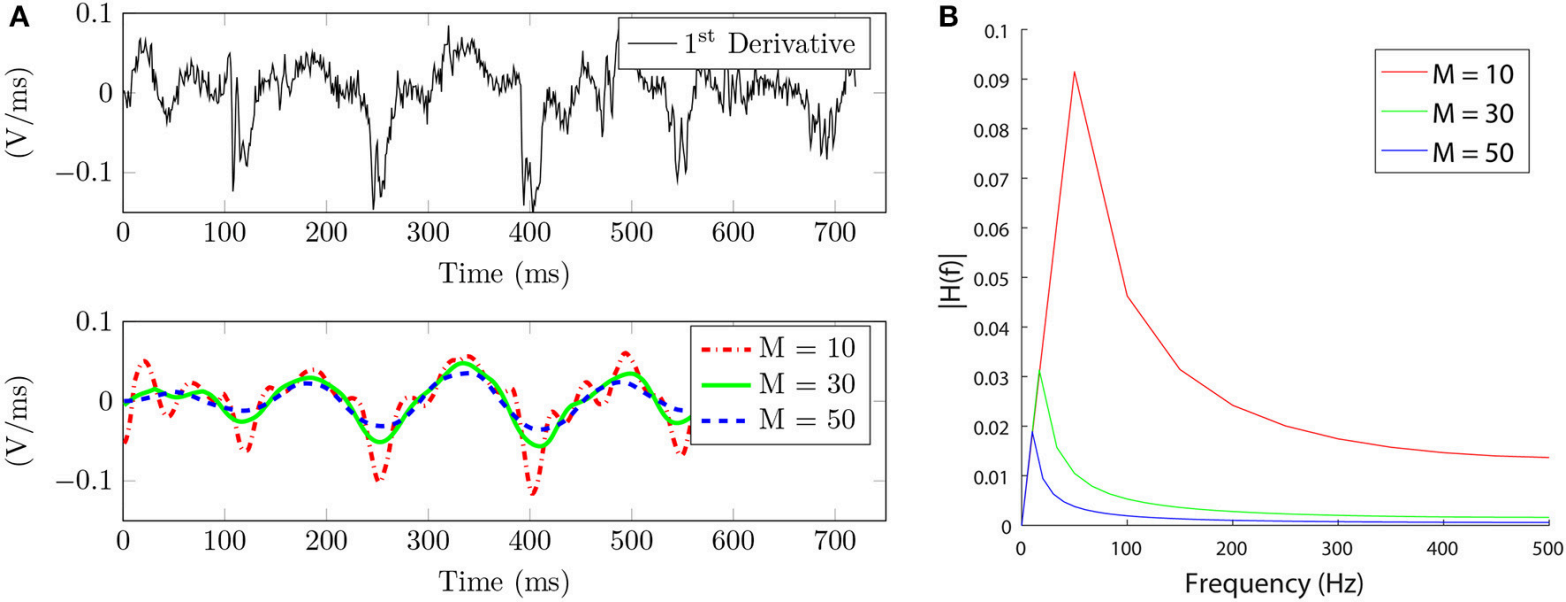
Signal slope characterization. **(A)** Top. Unipolar signal first derivative. Bottom. Unipolar signal slope approximation for different window length values, M in ms. **(B)** Frequency response of the equivalent filter *h*[*n*] in Equation (6) for different *M* values.

Additionally, we characterize the linear pattern approximation, which applied to EGMs resembles the outcome of a first derivative operator multiplied by the constant that depends on the window size *M*. The expression in Equation (5) can be seen as a filter with impulse response:

(6) $$\begin{array}{l} h\left[n\right]=M\cdot \overset{M}{\underset{\tau =-M}{\sum }}\tau \cdot \delta \left[n-\tau \right], \end{array}$$

and frequency response *H*(*f*) (Figure 4B), equivalent to a discrete-time low pass differentiator. In section Results, we analyze the effects of selecting the window length *M* on the amplitude and frequency components of the signal.

In the following, and since there is no ambiguity, we shall drop the subindex in the *n*_0_ and simply refer to it as *n*. The signal β[*n*] (Figure 2D) is inverted and rectified obtaining a new signal β^+^[*n*] (Figure 2E). The inversion pairs deflections with positive peaks of the signal, and the rectification discards the atrial components with positive slope. Then, the position of the positive peaks are assumed to correspond to the LATs.

The amplitude of the β^+^[*n*] peaks varies from activation to activation. These deviations are a consequence of the constant heart activity that prevents the electrodes from having a uniform atrial contact, resulting in amplitude changes. An exponentially decaying threshold after a peak detection is implemented to search for the local activation times (Barbaro et al., 1998). The threshold *T*_*h*_[*n*] is updated at each time instant as:

(7) $$T_{h}[n]=\{\begin{array}{ll} (M_{i}-\sigma )e^{-\frac{M_{i}-\sigma }{\tau }(n-(n_{i}+b))}+\sigma , & \text{if }n>n_{i}+b,\\ M_{i}, & \text{if }n_{i}<n\leq n_{i}+b. \end{array}$$

Variables *M*_*i*_ and *n*_*i*_ are the amplitude and time instant of the last detected peak. Time variable τ defines the decay rate of the exponential function. The constant σ specifies a lower limit for the threshold, filtering out small interferences or weak far-field contributions. The threshold is initialized to *M*_0_ = σ. Close depolarizations are physiologically improbable due to the refractory period of the tissue. To avoid false positives in this period, the threshold maintains its value for a blank period *b* before detecting a new peak. The algorithm detects a local activation time when a peak of the signal is above the threshold value, as Figure 2E shows. A local search for possible undetected peaks in a window of length *b* around each detected peak prevents false local maxima to be considered as activation instants.

### 2.5. Interpolation

In the atrium, depolarization flanks propagate locally as a consequence of the ionic sodium currents firing the cardiac cells through neighboring atrial tissue. We use signal spatial interpolation to represent the atrial activity in the area covered by the catheter. The subsequent processing thus becomes independent of the electrode topology employed. We have several potential magnitude candidates for interpolation: unipolar signals, local activations, and unipolar slopes. From the interpolation candidates, unipolar signals after ventricle cancellation produce noisy and ambiguous maps. The noise component inherent in the unipolar signal affects the LATs outcome, therefore performing an interpolation with noisy data is not recommended and should be avoided. Local activation times using binary signals (value 1 for LATs and 0 otherwise) offer poor discontinuous representation when recovering wavefronts. This interpolation does not preserve the dynamics of the wavefront since the nodes are only active one time instant corresponding to the LAT of the electrode associated to the grid node. To overcome these potential issues we choose to interpolate the signal β[*n*]. It provides a continuous transition, noise reduction, and contains information about the activation measures, coherent with the depolarization propagation basis in the atrium.

For the interpolation, we use a 2 dimensional (2D) squared grid consisting of *J* × *J* nodes, namely *n*_*j,k*_ with *j, k* ∈ [1, *J*], representing the atrial tissue covered by the catheter. The grid size is also related to the spatial resolution, e.g., for a PentaRay catheter range of 32 mm and *J* = 32 each node represents 1 mm^2^. In the grid, each β^(*i*)^[*n*] signal is mapped to the fixed spatial coordinate proportional to their location in the physical catheter, referring to these information nodes as *n*^(*i*)^. All these nodes belong to the set N. Grid nodes containing no signal are filled by means of interpolation using Shepard's method (Shepard, 1968). This interpolation technique uses inverse distance weighting to find an interpolated value based on the signals β^(*i*)^[*n*] for *i* = 1, …, *N*, and their node positions in the grid *n*^(*i*)^. The function for obtaining the interpolated signal β_(*j, k*)_[*n*] is defined as:

(8) $$\beta_{\left(j,k\right)}[n]=\left\{ \begin{array}{l} \beta^{\left(i\right)}[n],\text{ if }n_{\left(j,k\right)}\in N,\\ \frac{\sum_{i=1}^{N}w^{\left(i\right)}(n_{\left(j,k\right)})\;\cdot \;\beta^{\left(i\right)}[n]}{\sum_{i=1}^{N}w^{\left(i\right)}(n_{\left(j,k\right)})},\text{ otherwise}, \end{array}\right.$$

where

(9) $$\begin{array}{l} w^{\left(i\right)}\left(n_{\left(j,k\right)}\right)=\frac{1}{d{\left(n_{\left(j,k\right)},n^{\left(i\right)}\right)}^{p}}, \end{array}$$

being *d*(·, ·) any distance metric operator and *p* a positive real number power parameter. We use the Euclidean distance between the node positions since we employ two dimensional coordinates. In presence of 2D data, choosing *p* ≤ 2 causes the interpolated data to be dominated by far away points, so choosing *p* = 3or4 provides a better interpretation of local region information. After the interpolation, we apply LATs detection to all the grid nodes. While electrode positions are assumed to be not known in our method, Equations (8) and (9) can be easily adapted to reflect variable electrode position dependency in time and update the grid positions accordingly.

The interpolation method necessarily needs reliable ground truth data to prove its efficacy. For this reason we validated the spatial interpolation using *in silico* signals generated from a realistic atrial 3D model developed at the Karlsruhe Institute of Technology (Seemann et al., 2006). The model implements fiber orientation, spatial heterogeneities, and anisotropy conduction for both conduction velocity and ionic currents. Simulations were performed as in a previous study (Sánchez et al., 2017), with the AF-remodeled version of the cellular model by Maleckar et al. (2009). Simulations were run with the software Elvira (Heidenreich et al., 2010), and unipolar pseudo-electrograms (pEGMs) were calculated at each of these electrodes (Baher et al., 2007). The integration time step for the 3D atria simulations was 0.04 ms to properly generate the fast upstrokes of the action potential. The output voltages were post-processed every 1 ms to match the real AF signal acquired at a sampling frequency of *f*_*s*_ = 1 KHz.

**Figure 5:**
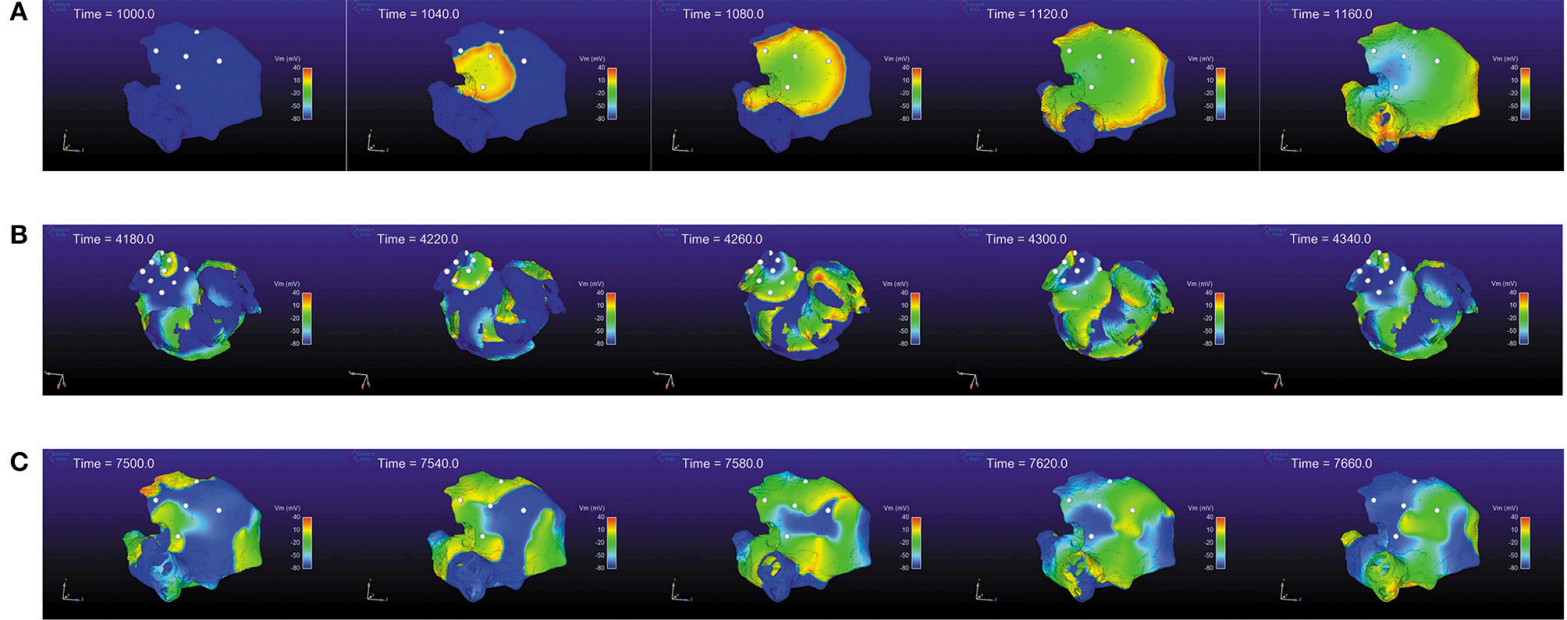
*In silico* simulation scenarios. **(A)** Sinus rhythm, 500 ms period. **(B)** Rotor. **(C)** Chaotic wavefront collisions.

In the *in silico* simulations (Figure 5), we considered different activity scenarios: sinus rhythm, rotor, and chaotic wavefront collisions exhibiting different propagation directions. The stimulation protocol applied a periodic stimuli for the sinus rhythm case, and forced extra-stimuli to generate reentries and fibrillatory behavior for the other cases. The recording positions in the atrium were manually chosen for the three scenarios. We deployed a squared 16 × 16 node grid, emulating 256 different electrodes recording the pEGM and AP signals. The grid was seized to fit the same area as the one covered with a PentaRay catheter. We simulated 10 s at a sampling frequency *f*_*s*_ = 1 KHz. We include the simulation videos as Supplementary Material.

### 2.6. Rotational activity detection

**Figure 6:**
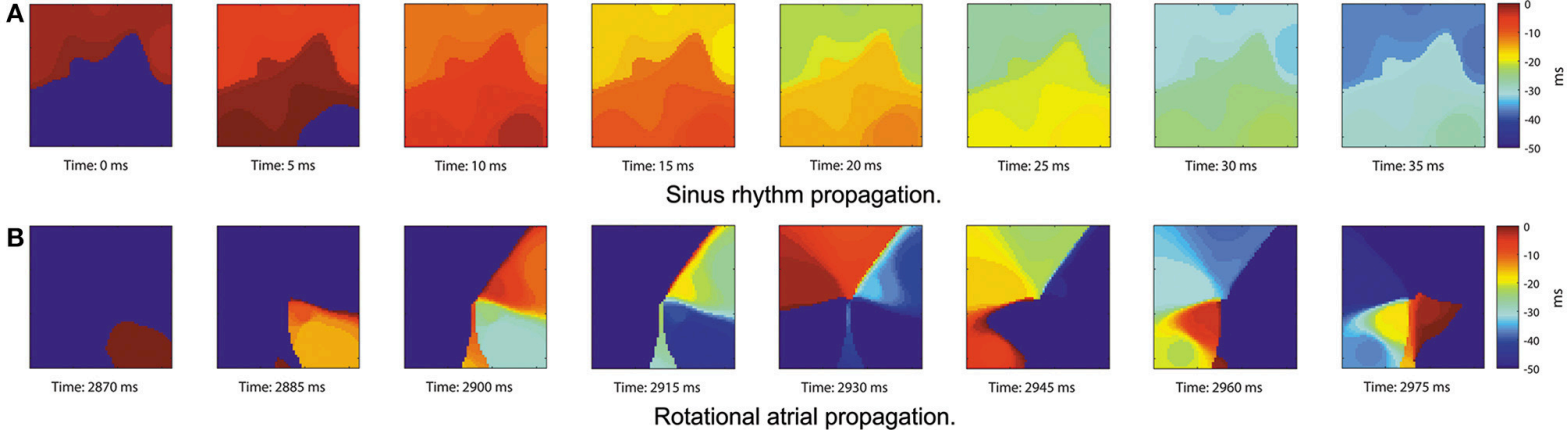
Real data propagation. Signals acquired with a PentaRay catheter at 8 different time instants showing the thresholded grid in the [−*P*, 0] ms range when a wavefront propagates, *P* = 50 ms. **(A)** Sinus rhythm activity propagating from top to bottom. **(B)** Propagation exhibiting counterclockwise rotational activity.

From the LATs, we represent the activations on the grid using isochronal maps (Ideker et al., 1989). The map takes value 0 when the node is active and linearly decrements its value until next activation occurs. The last *P* previous time instants of the signal are displayed (e.g., *P* = 40 ms), with smaller intervals producing narrower wavefronts. The most recent activated node will take a hotter color (red) and will cool down as time passes (blue) until a new activation occurs. Figure 6 provides an example of isochronal maps for *P* = 50 ms.

**Figure 7:**
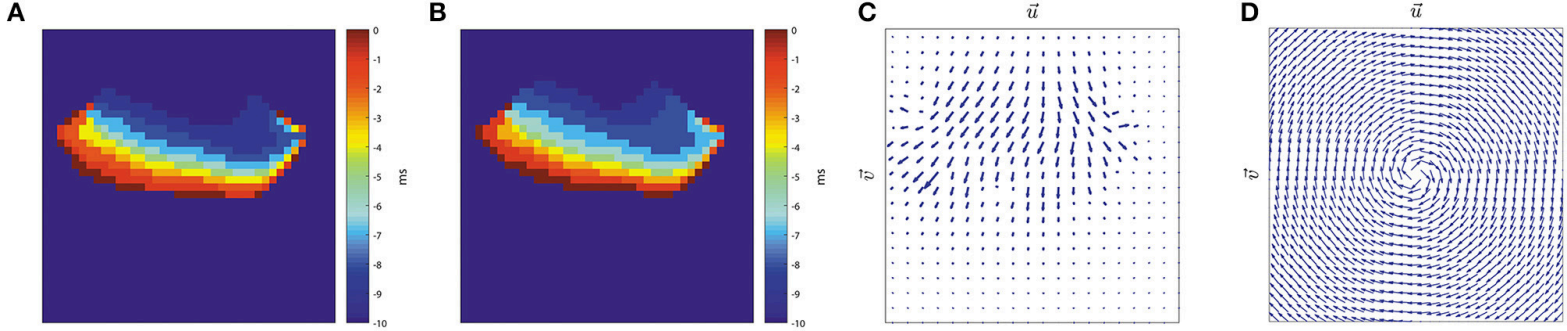
Optical flow. Horn-Schunck method on two consecutive images (Horn and Schunck, 1981). **(A)** Isochronal frame at *t* − 1. **(B)** Isochronal frame at *t*. **(C)** Velocity vectors applying Horn-Schunck method to the frames. **(D)** Clockwise rotation mask, grid size *J* = 32 nodes.

We propose to detect the presence of rotational activity on the isochronal maps estimating their optical flow (Ogle, 1951). Given two consecutive images (Figures 7A,B), it returns the velocity vectors $\vec{u}$ and $\vec{\upsilon }$ based on the difference of the two images, providing the propagation direction of the atrial wavefronts at each grid node, as Figure 7C shows.

Defining image intensity $I\left(\vec{x},t\right)$ as a function of time *t* and space $\vec{x}={\left[x,y\right]}^{T}$, the intensity translation can be expressed as:

(10) $$\begin{array}{l} I\left(\vec{x},t\right)=I\left(\vec{x}+\vec{r},t+1\right), \end{array}$$

where $\vec{r}={\left[u,v\right]}^{T}$ is the 2D velocity vector. In our case, $I\left(\vec{x},t\right)$ corresponds to the elapsed time since an activation occurred at node *n*_*j,k*_. Although many estimation approaches exist in the literature (Fleet and Weiss, 2006), an early method proposed by Horn and Schunck is used, based on non-parametric motion models and assuming smoothness in the whole image flow (Horn and Schunck, 1981). They proposed an energy functional for the flow:

(11) $$E(\vec{r})=\iint ((\nabla I\cdot \vec{r}+I_{t}{)}^{2}+\lambda (\|\nabla u{\|}^{2})+\|\nabla v{\|}^{2}))dxdy.$$

The solution to this equation can be iteratively computed for *u* and υ, the two components of the velocity vector $\vec{r}$. It obtains the partial derivatives *f*_*x*_, *f*_*y*_, and *f*_*t*_ by 2D-convolution of the images *I*_*t*_ and *I*_*t*−1_ with respect to the convolution kernels *K*_*x*_, *K*_*y*_ and *K*_*t*_, namely:

(12) $$\begin{array}{lll} f_{x} & = & I_{t}*K_{x}+I_{t-1}*K_{x}, \end{array}$$

(13) $$\begin{array}{lll} f_{y} & = & I_{t}*K_{y}+I_{t-1}*K_{y}, \end{array}$$

(14) $$\begin{array}{lll} f_{t} & = & I_{t}*K_{t}-I_{t-1}*K_{t}, \end{array}$$

where the convolution kernels are:

(15) $$K_{x}=\frac{1}{4}\left[\begin{array}{cc} -1 & 1\\ -1 & 1 \end{array}\right],$$

(16) $$K_{y}=\frac{1}{4}\left[\begin{array}{cc} -1 & -1\\ 1 & 1 \end{array}\right],\text{ and}$$

(17) $$K_{t}=\frac{1}{4}\left[\begin{array}{cc} 1 & 1\\ 1 & 1 \end{array}\right].$$

Values *u* and υ are approximated by iteratively calculating *N* times the solutions for

(18) $$\begin{array}{l} u^{n}={ū}^{n}-\frac{f_{x}\left[\left(f_{x}\cdot {ū}^{n}\right)+\left(f_{y}\cdot {\overset{-}{\upsilon }}^{n}\right)+f_{t}\right]}{\alpha^{2}+f_{x}^{2}+f_{y}^{2}}, \end{array}$$

(19) $$\begin{array}{l} \upsilon^{n}={\overset{-}{\upsilon }}^{n}-\frac{f_{y}\left[\left(f_{x}\cdot {ū}^{n}\right)+\left(f_{y}\cdot {\overset{-}{\upsilon }}^{n}\right)+f_{t}\right]}{\alpha^{2}+f_{x}^{2}+f_{y}^{2}}, \end{array}$$

for *n* = 1, …, *N* and *u*^0^ = 0, υ^0^ = 0, where α is the smoothing factor and the local averages ū^*n*^ and ${\overset{-}{\upsilon }}^{n}$ are calculated as:

(20) $$\begin{array}{lll} {ū}^{n} & = & u^{n-1}*\overset{-}{K}, \end{array}$$

(21) $$\begin{array}{lll} {\overset{-}{\upsilon }}^{n} & = & \upsilon^{n-1}*\overset{-}{K}, \end{array}$$

with the averaging kernel:

(22) $$\bar{K}=\left[\begin{array}{ccc} \frac{1}{12} & \frac{1}{6} & \frac{1}{12}\\ \frac{1}{6} & 0 & \frac{1}{6}\\ \frac{1}{12} & \frac{1}{6} & \frac{1}{12} \end{array}\right].$$

This method to approximate the integration and derivatives allows a large system of linear equations to be solved by iterative computation. We found *N* = 25 iterations to be enough to approximate the derivatives, since larger values produced almost identical results. The regularization parameter α provides global smoothing on the grid. For the computations, we selected a value α = 1 which makes the propagation of information over far distant points in the image possible.

We apply the HS method to the reconstructed wavefront images at each time instant, obtaining a two element vector ${\vec{r}}_{j,k}={\left[u_{j,k},v_{j,k}\right]}^{T}$ for each node. Vectors are normalized so $|{\vec{r}}_{j,k}|=1$, and ${\vec{r}}_{j,k}={\left[0,0\right]}^{T}$ if there is no propagation.

We introduce a circular pattern, see Figure 7D, consisting of unitary vectors ${\vec{c}}_{j,k}={\left[d_{j,k},e_{j,k}\right]}^{T}$ arranged in a spiral-like layout satisfying:

(23) $${\vec{c}}_{j,k}=\{\begin{array}{ll} d_{j,k}=sin(\alpha_{j,k}+\frac{\pi }{2}), & \text{for }j,k\in [1,J],\\ e_{j,k}=cos(\alpha_{j,k}+\frac{\pi }{2}), & \text{for }j,k\in [1,J], \end{array}$$

where α_*j,k*_ is the angle defined by each node *n*_*j,k*_ and the pattern center located at $n_{j^{*},k^{*}}$ calculated as:

(24) $$\alpha_{j,k}=atan2(\frac{d(j,j^{*})}{dist(k,k^{*})}),$$

where in this case the distance operator *d*(*A, B*) (introduced in Equation 9) stands for the euclidean distance between two points *A* and *B*.

This layout serves as comparison mask to quantify the rotation level. Vector velocity components of the wavefront and the reference mask are split into matrices [**U**, **V**] and $[\hat{U},\hat{V]}$ respectively. We apply element-wise scalar product at each time instant n to calculate the rotational intensity, normalized with respect to the number of nodes constituting the J-squared grid, as:

(25) $$\begin{array}{lll} T\left[n\right] & = & \frac{1}{J^{2}}\overset{J}{\underset{i=1}{\sum }}\overset{J}{\underset{j=1}{\sum }}u_{j,k}\left[n\right]d_{j,k}\left[n\right]+\upsilon_{j,k}\left[n\right]e_{j,k}\left[n\right]. \end{array}$$

The following step performs time integration of *T*[*n*] in a range of γ samples. Only values contained in the time interval [*n* − γ, *n*] are included to capture the wavefront dynamics, obtaining the expression for our new indicator

(26) $$\begin{array}{l} \Gamma \left[n\right]=\overset{n}{\underset{\tau =n-\gamma }{\sum }}T\left[\tau \right]. \end{array}$$

**Figure 8:**
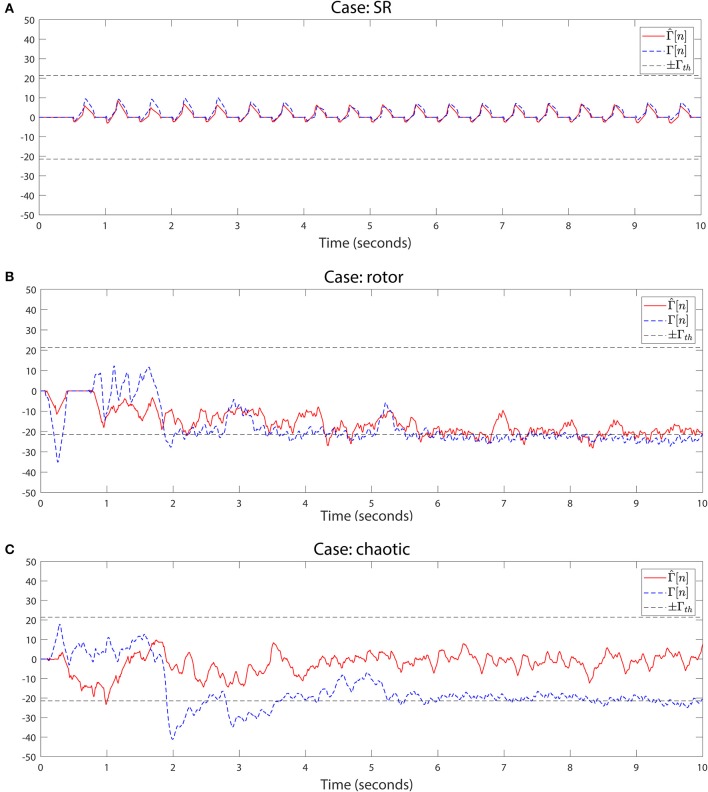
Rotational activity detector in *in silico* signals. Detection performed on the three simulation scenarios. The method detects rotational activation if the value of Γ[*n*] exceeds the upper threshold +Γ_*th*_ or falls below the lower threshold −Γ_*th*_. The sign of Γ[*n*] reflects the rotational gyre direction, being positive if the gyre matches the rotation mask spin (clockwise/counterclockwise depending on the chosen pattern), or negative if the propagation rotates in the opposite mask direction. For the simulation cases we applied the detection on the full Γ[*n*] and the interpolated $\hat{\Gamma }\left[n\right]$ grids to compare both outcomes. Signals from top to bottom: **(A)** Sinus rhythm. **(B)** Rotor. **(C)** Chaotic wavefront collision. Parameters were γ = 150 samples and Γ_*th*_ = γ/7.

**Figure 9:**
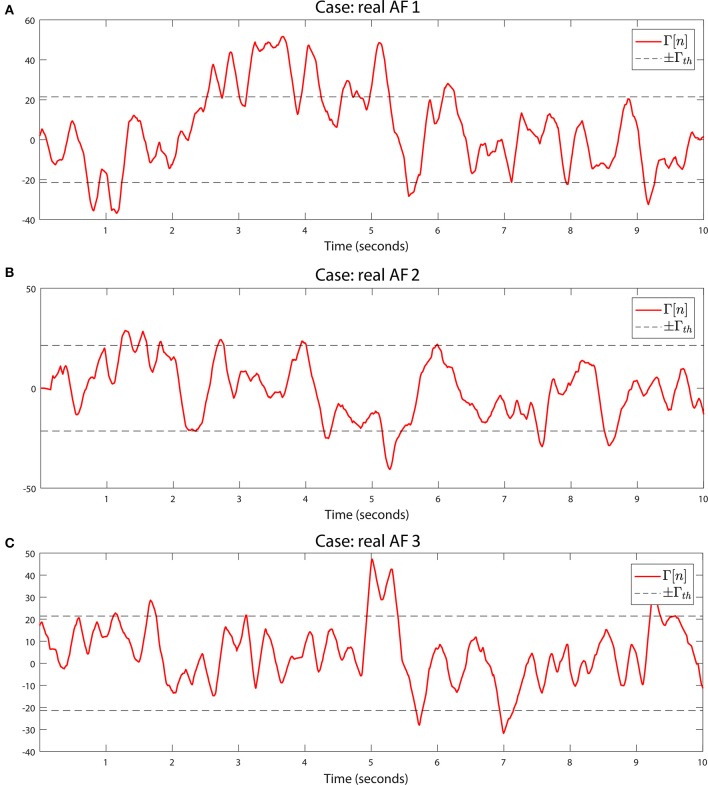
Rotational activity detector in real AF signals. Detection performed on the three real AF signals from three different patients. Same methods and parameters are applied as in Figure 8. **(A)** Sustained multiple rotational activation example. **(B)** Non-sustained multiple gyre rotations. **(C)** Another example of rotation detection.

We detect rotational activity when Γ[*n*] exhibits high or low values above or below a double decision threshold ±Γ_*th*_, see Figures 8A–C, 9A–C. The sign determines the circular direction of the wavefront, clockwise or counterclockwise. Depending on the chosen spin turn of the reference mask, i.e., if clockwise, positive Γ[*n*] peaks correspond to clockwise rotations and negative values to counterclockwise gyres.

### 2.7. Real-time implementation

The system (patent pending) is currently implemented in real-time using an ADC manufactured by National Instruments and a PC (Figure 1A). The dynamic range of the amplifier is ±2mV for intracavitary EGMs, and ±5mV for ECGs, with a voltage gain of 60 dB at the ADC input. Wilson central terminal acts as the indifferent electrode in unipolar configuration. Signals are filtered using a 50 Hz Notch filter, band pass filtered 0.05–50 Hz for unipolar EGMs, and 0.05–100 Hz for ECG leads. The ADC connected to the amplifier works at a sampling frequency of 1 KHz.

The computer has one 8-core Intel® Xeon® CPU 3.40 GHz processor, 16 GB RAM, on 64 bits Windows 7 Professional. A GPU, Nvidia Tesla K20c with 2496 CUDA cores, parallelizes the computation minimizing the time for delivering rotational activity detection.

The ventricle cancelation method requires to buffer signals long enough to capture several ventricle beats, we buffer the last *B* seconds of the signals to estimate each channel ventricular pattern, e.g., *B* = 10 s. Computations take less than 7 s for *B* = 10 s, and a screen inside the operating room presents the results immediately. Wavefront propagation intervals where the value of Γ[*n*] exceeds the detection threshold are presented to the electrophysiologist in a monitor at reduced speed, as Figure 1B shows. Then, the rotor location in the atrium can be annotated in any 3D electroanatomical mapping system as guiding reference during ablation.

## 3. Results

### 3.1. Local activation times results

We evaluated the LATs detection in real AF and computer model EGMs. We validated our method by direct look at the EGMs and LATs outcomes with expert knowledge from electrophysiologists and fine adjustment of the parameters taking into account noise reduction, electrode adjacency to maximize the detection of activations (for both real and simulated signals). We considered a range of 20−40 ms as the span of a depolarization, which corroborates previous results (Yue et al., 2005; Narayan et al., 2008).

We used the unipolar signal in Figure 3C to analyze the filtering effect of parameter *M* comparing it with the unipolar signal first derivative used by other authors to identify LATs. Our method provides a smoother and less noisy signal than the one obtained with the first derivative, see Figure 4A. This makes it easier to identify the LATs, where our method presents high ${\hat{\beta }}_{MSE}\left[n\right]$ values when the pattern matches a deflection.

In terms of frequency effects, small values of *M* produce a higher cut-off frequency, whereas big values of *M* present a more restrictive low pass characteristic which filters more noise, see Figure 4B. This is helpful in noisy EGMs, where small residual peaks appear, and complementary, the exponential decaying threshold demonstrated to recover from false high peaks. The steep threshold drop avoids LAT error propagation that would lead to miss the next atrial activations. Value τ was set empirically to τ = 3.5·10^−3^ by reviewing atrial activations. We also found threshold parameter *b* crucial in the event of fragmented EGMs, by adjusting the blank period to several ms (60 ms) the fragmented activity can be detected and taken into account in the reconstructed wavefront. This value was selected to be above the classical dominant frequency (DF) range of 4−9 Hz shown by most studies (Ropella et al., 1988). On the other hand, higher values would not allow the threshold to decay fast enough for the next activation to be detected, missing LATs. The value of *b* = 60 ms was also proposed according to the closeness of consecutive atrial activations and the recovery time for the exponential threshold to reach its minimum value σ. When the next activation exhibited smaller peak amplitude, values greater than 60 ms incurred in missing atrial activations, since the threshold was not fast enough to decay and identify the β^+^[*n*] positive peak. We tried different values for *b* and finally selected *b* = 60 ms.

In terms of amplitude, the window selection has a direct effect on the amplitude of the filtered signal. From Figure 4A (bottom) we can see this behavior as the value of the window *M* is increased. The signal becomes softened at the expense of reducing its amplitude. The same interpretation is derived from the frequency response analysis in Figure 4B, where the amplitude of the frequency spectrum becomes reduced as *M* increases.

After analyzing the effect on the window length selection, we can conclude that employing an over-sized window length would lead the peaks to be less well defined because the signal becomes flatter, but as an advantage unipolar noise is greatly reduced. On the other hand, having a window too narrow (small *M*) would produce sharper transitions but would be more sensitive to small deflections that might be miss-detected as LATs. From the *in silico* simulation scenarios used to validate the methods, see Interpolation subsection, Figure 5, we found that a value of *M* = 20 − 30 samples reports a good trade-off between signal amplitude and noise reduction.

**Figure 10:**
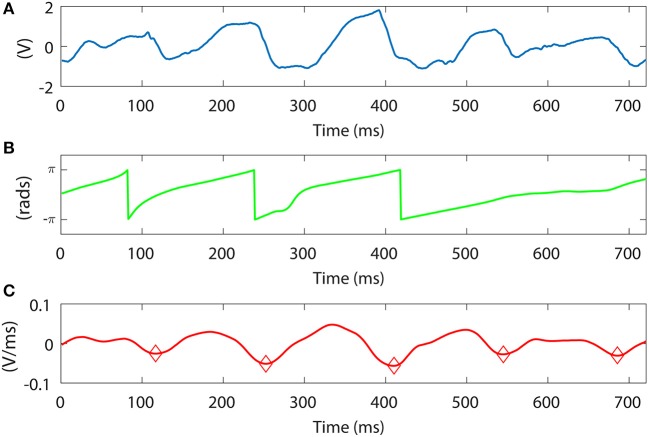
Signal slope and Hilbert transform phase comparison. **(A)** Unipolar signal. Bottom. Unipolar signal slope approximation for different window length values, M in ms. **(B)** Hilbert transform phase applied to the signal in **(A)**. **(C)** Unipolar slope approximation applied to the signal in **(A)**, *M* = 30 ms. Red circles denote the LATs calculated with our method.

Furthermore, we want to compare our LAT detection method vs. the phase signal obtained with the Hilbert transform (Kuklik et al., 2016). One representative example comparing both methods is shown in Figures 10A–C, using the same unipolar signal as in Figure 3C. In the example, our method is able to capture all the activation time instants, denoted as diamonds Figure 10C. We can clearly identify the missing phase transition at the 4th and 5th atrial activations. In this situation the Hilbert transform is not reliable, confirming previous results on weak correlation between phase and activation maps (Vijayakumar et al., 2016). Moreover, the Hilbert transform requires an estimation of the signal period, calculated as the dominant frequency of the segment which can vary in time. Additionally in Figure 10B we can see how the first phase transition drops from π to −π preceding the first two activations (34 ms and 13 ms respectively), and after them it gets delayed (10 ms) in the third activation. This is a meaningful variation that may lead to a potential misinterpretation of the atrial activity. Since our method relies on beat to beat detection it does not shift the LATs from activation to activation.

### 3.2. Signal interpolation results

We used *in silico* signals to validate the wavefront reconstruction. From the 256 pEGM signals, we chose 20 signals according to the spatial position of the PentaRay electrodes in the grid. We obtained their slope information β[*n*], performed the interpolation and the LATs detection to obtain the reconstructed wavefronts. To evaluate the outcome of the interpolation method, we also processed the whole 256 pEGMs grid to obtain the isochronal propagation so we could compare both interpolations.

We evaluated the interpolation reconstruction for different *M*, *p* and σ parameter values. We quantified the interpolation performance by measuring the LATs relative root mean square error (rRMSE) between the interpolated and full grid versions for all the parameter combinations. The rRMSE is defined as:

(27) $$\begin{array}{l} rRMSE=\frac{\sqrt{\frac{1}{N}\overset{N}{\underset{n=1}{\sum }}\overset{J}{\underset{i=1}{\sum }}\overset{J}{\underset{j=1}{\sum }}{\left(L_{i,j}\left[n\right]-{\hat{L}}_{i,j}\left[n\right]\right)}^{2}}}{\sqrt{\frac{1}{N}\overset{N}{\underset{n=1}{\sum }}\overset{J}{\underset{i=1}{\sum }}\overset{J}{\underset{j=1}{\sum }}{\left(L_{i,j}\left[n\right]\right)}^{2}}}, \end{array}$$

where *L*_*i,j*_[*n*] and ${\hat{L}}_{i,j}\left[n\right]$ are the full grid and interpolated LAT signals at node *n*_*i,j*_ and time instant *n* ∈ [1, *N*] samples. The *L*_*i,j*_[*n*] and ${\hat{L}}_{i,j}\left[n\right]$ take value 0 when a LAT is detected and linearly decrease their values until a new activation occurs. We used value ranges *M* = 2, 3, 4, …, 20, *p* = 1, 2, 4 and σ = 0.05, 0.06, …, 0.29, 0.30. As Supplementary Material we include the videos generated for the three simulation scenarios (sinus rhythm, rotor, and chaotic wave collisions) comparing the full and interpolated grids for the values minimizing the rRMSE.

**Figure 11:**
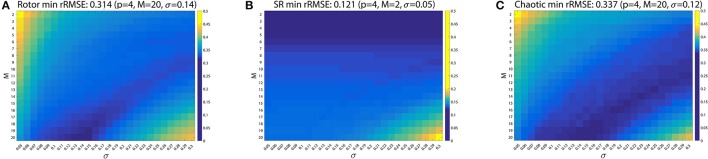
Shepard's interpolation method performance. The relative RMSE (rRMSE) is represented for each case for *p* = 4. **(A)** Rotor. *rRMSE* = 0.314 for *p* = 4, *M* = 20 and σ = 0.14. **(B)** Sinus rhythm. *rRMSE* = 0.121 for *p* = 4, *M* = 2 and σ = 0.05. **(C)** Chaotic. *rRMSE* = 0.337 for *p* = 4, *M* = 20 and σ = 0.12.

Best error results were achieved for *p* = 4 in all cases. Figure 11 shows the rRMSE values for the *M* and σ combinations for *p* = 4. For the rotor case (Figure 11A) the value minimizing the reconstruction error was *rRMSE* = 0.314 (rRMSE mean ± std, 0.348 ± 0.025) achieved for *p* = 4, *M* = 20 and σ = 0.14. Sinus rhythm (Figure 11B) scored *rRMSE* = 0.121 (0.131 ± 0.011) for *p* = 4, *M* = 2 and σ = 0.05, and the wave collision case (Figure 11C) *rRMSE* = 0.337 (0.357 ± 0.019) for *p* = 4, *M* = 20 and σ = 0.12.

The interpolation using Shepard's method was compared against bilinear interpolation. Statistical significance analysis was perform, comparing the rRMSE group means and standard deviations using the Kruskal-Wallist test. Significance was considered for a two-sided *p*-value (*p*) of less than 0.01. The minimum rRMSE for rotor case was *rRMSE* = 0.328 (0.375 ± 0.026) for *M* = 20 and σ = 0.14 (same parameters as the Shepard's approach). The SR case achieved *rRMSE* = 0.120 (0.126 ± 0.012) for *M* = 6 and σ = 0.28, and the chaotic case *rRMSE* = 0.362 (0.401 ± 0.026) for parameters *M* = 16 and σ = 0.21. In the rotor and chaotic cases the bilinear interpolation offered significant worse minimum error than the one obtained with the Shepard's interpolation method (*p* = 7.6 × 10^−59^ and *p* = 4.6 × 10^−110^ respectively). Only in the sinus rhythm case the bilinear interpolation was significantly better than the Shepard's method, *rRMSE* = 0.120 and *rRMSE* = 0.121 respectively (*p* = 4.6 × 10^−43^). Since the system is expected to operate when patients are in AF, Shepard's interpolation method provides better performance. We include the Supplementary Figure 1 for the three *in silico* scenarios rRMSE as Supplementary Material, analogous to Figure 11.

We now study the effect on the interpolation performance when a catheter branch differs from the fixed position in the grid for the three *in silico* cases. To characterize this behavior, we rotated the positions of one of the branches of the catheter, i.e., the PentaRay model, from its predefined position. We rotated the 4 electrodes in the branch covering a rotation range [−θ, θ], with $\theta =\frac{2\pi }{5}$ radians (or 72°), establishing as rotation limits the angle where the branch overlaps its two neighboring branches. At the rotated electrode positions we took the signals from the full simulated grid and used them to perform the interpolation on the fixed interpolation positions. This way the interpolation maintains the fixed electrode layout but the information signals come from shifted positions emulating the behavior of the catheter when a branch does not match the predefined layout. We iterated in steps of $\frac{\theta }{20}$ and calculated the rRMSE for all the rotated interpolations. We include the results in the Supplementary Figure 2. The figure shows the interpolation rRMSE for the three cases in the [−θ, θ] in radians and also with respect the length of the arc of the rotation angle. The arc length is calculated from the circumference of radius 32 mm determined by the most distant PentaRay electrode with respect to the center of the catheter. The rRMSE remains almost the same for a rotation of $\frac{\pi }{50}$ radians (±2 mm) and worsens as the rotation angle moves away from the reference fixed position.

The interpolated reconstructions for the parameters minimizing the rRMSE criterion in the three *in silico* cases were presented to the electrophysiologists for final validation. They agreed that the sinus rhythm and the rotor cases presented almost identical representation compared to the simulated ground truth. The wave collisions case also exhibited good results in the presence of chaotic behavior. In all the cases the interpolation managed to recover all the activations present in the pEGM grid, and the wavefront morphologies also matched the original ones. This evaluation supports the effectiveness of the proposed interpolation method, and discards any interpolation effect that may introduce uncertainty in the rotational activity detection system.

### 3.3. Rotational activity detection results

The lack of available rotor signals, not even being a consensus about their existence, presents a challenge when evaluating the system's ability to detect rotors. For this reason scientists resort to *in silico* simulated environments to test their methods. We applied the detection on the three *in silico* scenarios for the full and interpolated grids, with γ = 150 (150 ms window for *f*_*s*_ = 1 KHz), obtaining signals Γ[*n*] and $\hat{\Gamma }\left[n\right]$ respectively. As shown in Figures 8A–C, the interpolated and the full versions behave similarly when we capture their dynamics with the rotation mask. We set the same threshold Γ*th* = ±γ/7 for the three cases, which succeeded to completely detect the rotor simulation during the whole interval the spiral is active, Figure 8B. In the sinus rhythm case, Figure 8A, no rotational activity is detected, as expected from its homogeneous propagation. The chaotic wavefront collisions, Figure 8C triggers the detector at some points in the full grid Γ[*n*] signal. The randomness of the activation makes the wavefront to partially rotate around the grid center, but since the rotation is not sustained in time nor exhibits a complete turn it does not yield a false detection positive.

The results using the *in silico* simulations allowed us to adjust the system parameters to automatically detect rotational activity in real-time in patients. We acquired signals from 28 AF patients using the PentaRay catheter mapping different atrial areas per patient. We created a database with more than 600 registers containing EGM and ECG signals. We analyzed the EGMs and reconstructed wavefronts, and conclude that the *in silico* simulations threshold value of Γ*th* = γ/7 exhibited great detection performance for rotational activity in real AF signals. As detection examples, we include the Γ[*n*] signal of a rotational activation detected in three of the patients, Figures 9A–C. We acquired the signal at *f*_*s*_ = 1 KHz and used γ = 150 to match the same integration interval as the computer simulations.

In the first example, Figure 9A, Γ[*n*] captures the atrial activity as it performs multiple continuous gyres around the center in the 2–5 s interval. The video corroborating presence of the rotational activity is included in the Supplementary Material. The method also captures activations that exhibit single or incomplete gyres. A couple of not maintained rotations are also captured at the beginning and end of the acquisition, 0–1 s and 9–10 s intervals respectively. This is important since the gyre incompleteness can be related to areas in which the activation experiments a change of direction that may explain AF maintenance, or can be even related to meandering rotational activation. In the second example, Figure 9B, the wavefronts describe a multiple gyre between 1.2 and 1.8 s that evolves into some incomplete gyres, around 4.3, and 8.7 s. The incomplete gyre at 4.3 s precedes a complete gyre at 5.3 s. In the third example, Figure 9C, we show another case of multiple gyre detection at the 4.7–5.4 s interval which evolves into an incomplete gyre at time instant 5.74 s. The activity triggers again the detector at 7 s, and again detects a multiple gyre around the end of the acquisition, interval 9.2–9.5 s. With these examples we show the capability of the system to detect rotational activation concerning incomplete, complete and multiple gyres. The latter, exceeds the threshold for a more prolonged duration in time, which is useful to differentiate the complexity of the gyre.

**Figure 12:**
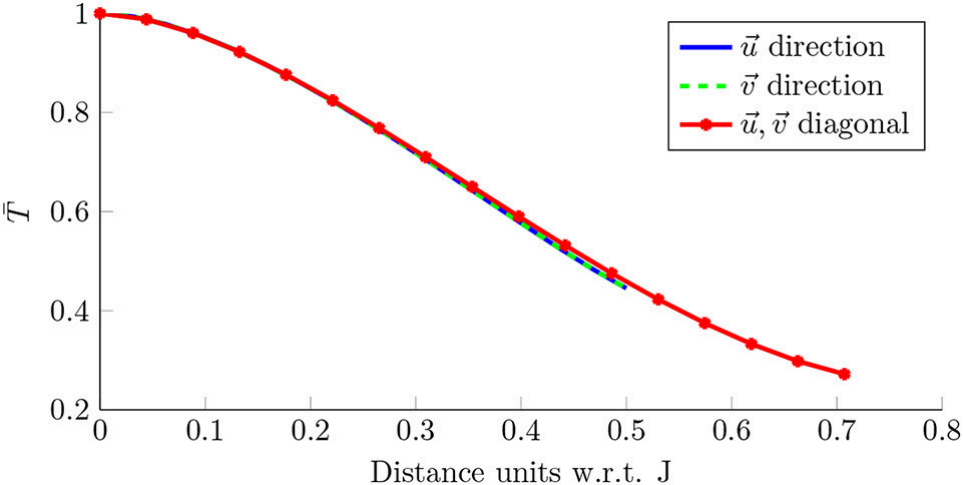
Detection robustness. Scalar product for the rotation mask in Figure 7D, when it is compared with its shifted version in the $\vec{u}$ axis (blue), $\vec{\upsilon }$ axis (green) and the $\vec{u}-\vec{\upsilon }$ diagonal (red). The shift is expressed in J units (nodes).

Additionally, the method is robust against non-centered rotational activations. We tested the rotational activity detection robustness against non-centered rotational activations. Figure 12 shows the scalar product *T*[*n*] (Equation 25) between the reference mask and its shifted version in the $\vec{u}$ and $\vec{\upsilon }$ axis, emulating a rotation whose center moves away from the origin. With no shift, the pattern overlaps itself and the scalar product is maximum. If the center of rotation moves further, $\overset{-}{T}$ decreases its value. For a distance of 0.3*J* nodes, the scalar product scores 70% of the centered pattern value, i.e., 10 nodes with *J* = 32 or 19 nodes with *J* = 64, capturing the rotational activity. The approximate *J* correspondence in mm attaining the PentaRay catheter coverage when it is fully deployed is 32 mm. That means that for a rotational activity mask shifted 0.3*J* we capture at least 70% of its dynamics at 9.6 mm, which extends the operative physical range of the detector.

## 4. Discussion

Catheter AF ablation was developed more than 20 years ago. Since its outbreak, this clinical technique obtained international acceptance among the clinicians. In the process with the development of new tools, it increased its safety and decreased the procedure duration. However, there is no perfect AF treatment procedure described yet, since our understanding of the AF mechanisms remains poor. In this way new ablation procedures are evolving with the technology, trying new strategies besides the standard isolation of the pulmonary veins.

Under these circumstances, we provide an assessment tool for evaluating the influence of rotational activity in AF. Although the presence of rotational activity is itself controversial, we individually validated each of the steps of the procedure and obtained evidence of the presence of rotational activity in AF patients.

We believe that technology and clinical practice must go hand in hand, as new and more sophisticated technological advances emerge every year. In this way, one of most demanding requirements is to deliver results in real-time. We integrate signal acquisition and processing in our system, which allows direct acquisition of the signals from the amplifier without requiring signal exportation from a recording device and additional post-processing (which introduce an unacceptable delay in the clinical procedure). We address the computational time handicap by designing parallelizable signal processing steps. We employ multi-core processors and GPU based code to distribute the computations and minimize the processing times, achieving near real-time results. The latency of the system can be further reduced by a combination of more fine-grained parallelization and custom hardware, allowing also the implementation of more complex algorithms and additional assessment tools.

We identify that LAT information in unipolar EGMs can be of potential value as an alternative to phase mapping analysis. More particularly, our EGM filtering and LAT detection approach emerges as an alternative for EGM signal processing, being extendable to other kind of biological signals, e.g., electroencephalograms, electromyograms, or galvanic skin response. The present pattern match, for rotational activation detection, allows other pattern layouts to be studied which could be of help in other studies. These patterns can be predefined at the beginning of the procedure or they can be learned by means of machine learning methods during signal acquisition.

*In silico* simulations need to be part of any new method development, as they provide validation tools, reproducibility and variety in controlled scenarios. The *in silico* signal simulations have been proven useful to validate the interpolation of the signals as prior step to reconstruct the propagation grid. This provided us with a framework to study how the selection of the different parameters affect the signal processing steps. Using a value of σ which is too large prevents the detection of LATs, while a large value of *M* also limits the LAT detection because the signal is low-pass filtered and attenuated. With the study of different parameter combinations, we reached a compromise between the noise reduction and the LAT detection which succeeded to minimize the error committed when interpolating the signals.

The unavailability of the electroanatomical 3D system to provide the real-time position of the electrodes forces our method to rely on a fixed layout. This constraint requires the electrophysiologist to operate the catheter and align it to the fixed electrode layout. But on the other hand, this ensures the electrode contact against the atrial wall, and helps to better interpret the behavior of local atrial areas complementary to other methods based on basket catheters. We analyzed the effect of a branch drift with respect to the reference layout, concluding that a shift of ±2 mm produces almost identical results as the correctly placed electrodes, Supplementary Figure 2. We note that some areas of the left atrium cannot be so easily mapped, since deployment of catheters sometimes presents a challenge even for experienced electrophysiologists. But this goes in parallel with basket catheters, whose geometry does not allow to access and map certain areas due to limited coverage and deformations of the catheter. Further studies on tissue characterization could relate atrial areas of restricted access with measurable indices, e.g., bipolar voltage or impedance values.

In this line, the relationship between the atrial sites presenting rotational activity in AF and its association with scar tissue was studied employing our system (Ruiz Hernandez et al., 2017). The complexity of rotational activity (i.e., if the rotation exhibits an incomplete, complete, multiple or no gyre) was compared with the scar value at the observed atrial sites (dense < 0.1 mV, non-dense [0.1, 0.5] mV, or non-scar [0.5, 1.5] mV). The results identified rotational activity locations not associated with any particular left atrial location, and significant positive correlation between the voltage (scar level) and the gyre complexity. This initial study offers promising results to characterize the areas that may be prone to anchor rotational activity but that are difficult to access with mapping catheters. In this sense, the system is currently being used in a new ablation strategy study, radial ablation. The study proposes to ablate the rotor locations and connect the rotor sites with the standard pulmonary vein isolation (PVI) ablation lines, as new studies in monolayer atrial tissue confirm (Feola et al., 2017). The purpose of this novel ablation strategy will assess if radial ablation reduces AF recurrence with respect to single PVI ablation, and it will help to improve the system with direct feedback from the electrophysiologists.

Nevertheless, further study is necessary for corroborating the validity of our approach. The rotor assessment using other catheter topologies in real-time, and the results of forthcoming studies will further validate the system and its inherent methods.

## Ethics statement

All patients gave full informed consent and the study was approved by the Institutional Review Board (IRB) of the center.

## Author contributions

All authors contributed equally to the conception and design of the study. ÁA and AA-R acted as supervisors of the research and contributed in methodological aspects. GR-M and ÁA acquired the signals from patients. GR-M and AA-R were responsible for performing the data analysis and developing the algorithms and methods. All authors contributed to manuscript revision, read and approved the submitted version.

### Conflict of interest statement

The authors declare that the research was conducted in the absence of any commercial or financial relationships that could be construed as a potential conflict of interest.

## Supplementary material

Supplementary Video 1 *In silico* sinus rhythm. The file contains the sinus rhythm simulation scenario with a single activation source every 500 ms emulating the normal activation pattern in the atrium.

Supplementary Video 2 *In silico* rotor. The file contains the rotor case where a clockwise activation spins around the grid center. After sinus rhythm and some collisions the rotor manages to anchor and remains stable in the grid location, exhibiting complete reentry rotations that are maintained in time.

Supplementary Video 3 *In silico* chaotic waves. The file focus on an area of chaotic wave collisions in the center of the atrium during simulated AF. The wavefronts collide and break forming in some cases rotor-like patterns that are not maintained in time.

Supplementary Video 4 *In silico* sinus rhythm interpolation. The file represents the pseudo-electrograms (pEGMs) propagation wavefronts vs. the best reconstruction for the sinus rhythm case (Figure 5A), obtained for the values, *M* = 2, *p* = 4, and σ = 0.05.

Supplementary Video 5 *In silico* rotor interpolation. The file represents the pEGMs propagation wavefronts vs. the best reconstruction for the rotor case (Figure 5B), obtained for the values *M* = 20, *p* = 4, and σ = 0.14.

Supplementary Video 6 *In silico* chaotic waves interpolation. The file represents the pEGMs propagation wavefronts vs. the best reconstruction for the wavefront collision case (Figure 5C), obtained for the values *M* = 20, *p* = 4, and σ = 0.12.

Supplementary Video 7 Real AF signal. The file contains the reconstructed wavefront of a rotor spinning multiple times. We acquired the signals during a live AF ablation procedure using a PentaRay catheter. The video shows 10 s of the atrial activity present in the acquired signals. Figure 9A represents the Γ[*n*] signal associated to the video were the rotational activity detection is performed. The rotational activity starts at 2,400 ms, where some reentry pattern is observed but not maintained. At 3,200 ms a multiple gyre appears spinning 4 times in a row. After this event some partial and one complete rotation is detected, but the rotation is not maintained in time as the previous multiple gyre.

Supplementary Figure 1 The figure shows the rRMSE for the bilinear interpolation applied to the three *in silico* scenarios, and serves as direct performance comparison to the Shepard's interpolation method in Figure 11. **(A)** Rotor. *rRMSE* = 0.328 for *M* = 20 and σ = 0.14. **(B)** Sinus rhythm. *rRMSE* = 0.120 for *M* = 6 and σ = 0.28. **(C)** Chaotic. *rRMSE* = 0.362 for *M* = 16 and σ = 0.21.

Supplementary Figure 2 The figure shows the rRMSE when the electrodes in a catheter branch are rotated an angle θ from the predefined interpolation position. The top x-axis covers the [−θ, θ] angle range for $\theta =\frac{2\pi }{5}$ radians (or 72°). The bottom x-axis is in linear units and represents the length of the rotated arc of the 32 mm circumference containing the most distal electrode of the PentaRay catheter. The figure displays the three *in silico* cases: rotor, SR and chaotic wavefronts. The rRMSE error remains almost identical for a shift of ± 2 mm.
