# Corrigendum: Real-Time Rotational Activity Detection in Atrial Fibrillation

**DOI:** 10.3389/fphys.2018.01260

**Published:** 2018-09-04

**Authors:** Gonzalo R. Ríos-Muñoz, Ángel Árenal, Antonio Artés-Rodríguez

**Affiliations:** ^1^Signal Theory and Communications Department, Universidad Carlos III de Madrid, Madrid, Spain; ^2^Gregorio Marañón Health Research Institute, Madrid, Spain; ^3^Department of Cardiology, Hospital General Universitario Gregorio Marañón, Madrid, Spain

**Keywords:** atrial fibrillation, multi-electrode catheter, signal processing, real-time, rotors, rotational activity

In the original article, we neglected to include the funder Beca de la Sección de Electrofisiología y Arritmias de la SEC. Therefore, the Funding section was updated:

This work has been partly supported by MINECO/FEDER (ADVENTURE, id. TEC2015-69868-C2-1-R), Comunidad de Madrid (project CASI-CAM-CM, id. S2013/ICE-2845), and the grant Beca de la Sección de Electrofisiología y Arritmias de la SEC.

We found a caption error in Figure 8, where labels A–C should be reordered to **(A)** Sinus Rhythm. **(B)** Rotor. **(C)** Chaotic wavefront collision, and the sign of the second reference to +Γ_*th*_ should be changed to −Γ_*th*_. The caption was updated to reflect this change:

**Figure 8**. Rotational activity detector in *in silico* signals. Detection performed on the three simulation scenarios. The method detects rotational activation if the value of Γ[*n*] exceeds the upper threshold +Γ_*th*_ or falls below the lower threshold −Γ_*th*_. The sign of Γ[*n*] reflects the rotational gyre direction, being positive if the gyre matches the rotation mask spin (clockwise/counterclockwise depending on the chosen pattern), or negative if the propagation rotates in the opposite mask direction. For the simulation cases we applied the detection on the full Γ[*n*] and the interpolated $\hat{\Gamma }\left[n\right]$ grids to compare both outcomes. Signals from top to bottom: **(A)** Sinus rhythm. **(B)** Rotor. **(C)** Chaotic wavefront collision. Parameters were γ = 150 samples and Γ_*th*_ = γ/7.

Additionally, the caption in the Figure 12 should reference the Figure 7D, and not Figure 3C.

The authors apologize for these errors and state that this does not change the scientific conclusions of the article in any way.

The original article has been updated.

## Conflict of interest statement

The authors declare that the research was conducted in the absence of any commercial or financial relationships that could be construed as a potential conflict of interest.

