# Supplementary figures and images for: Real-Time Rotational Activity Detection in Atrial Fibrillation
Source: Front Physiol. 2018 Mar 13;9:208. doi: 10.3389/fphys.2018.00208 (PMC5859379; doi:10.3389/fphys.2018.00208)

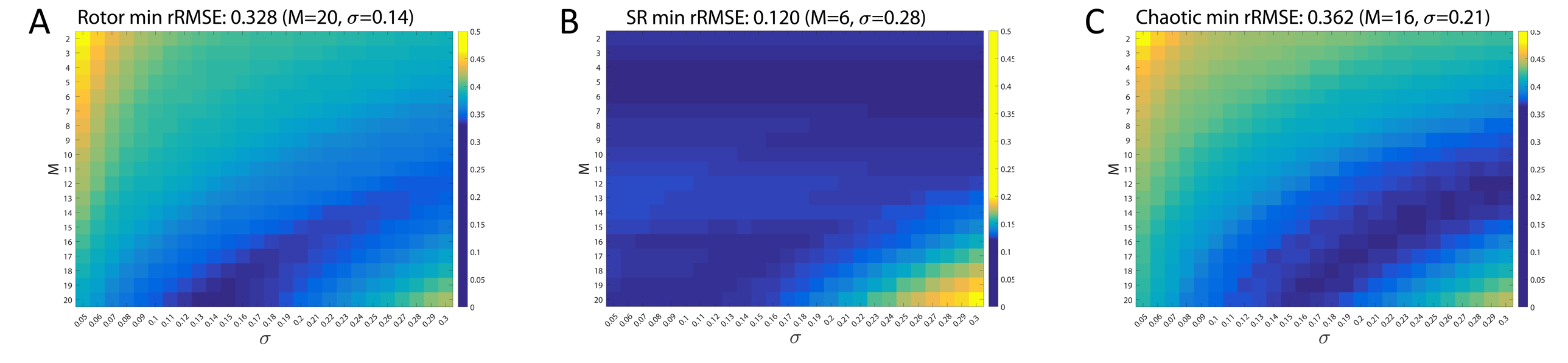

Supplement: Supplementary Figure 1 — The figure shows the rRMSE for the bilinear interpolation applied to the three in silico scenarios, and serves as direct performance comparison to the Shepard's interpolation method in Figure 11. (A) Rotor. rRMSE = 0.328 for M = 20 and σ = 0.14. (B) Sinus rhythm. rRMSE = 0.120 for M = 6 and σ = 0.28. (C) Chaotic. rRMSE = 0.362 for M = 16 and σ = 0.21. [file Image1.TIF]

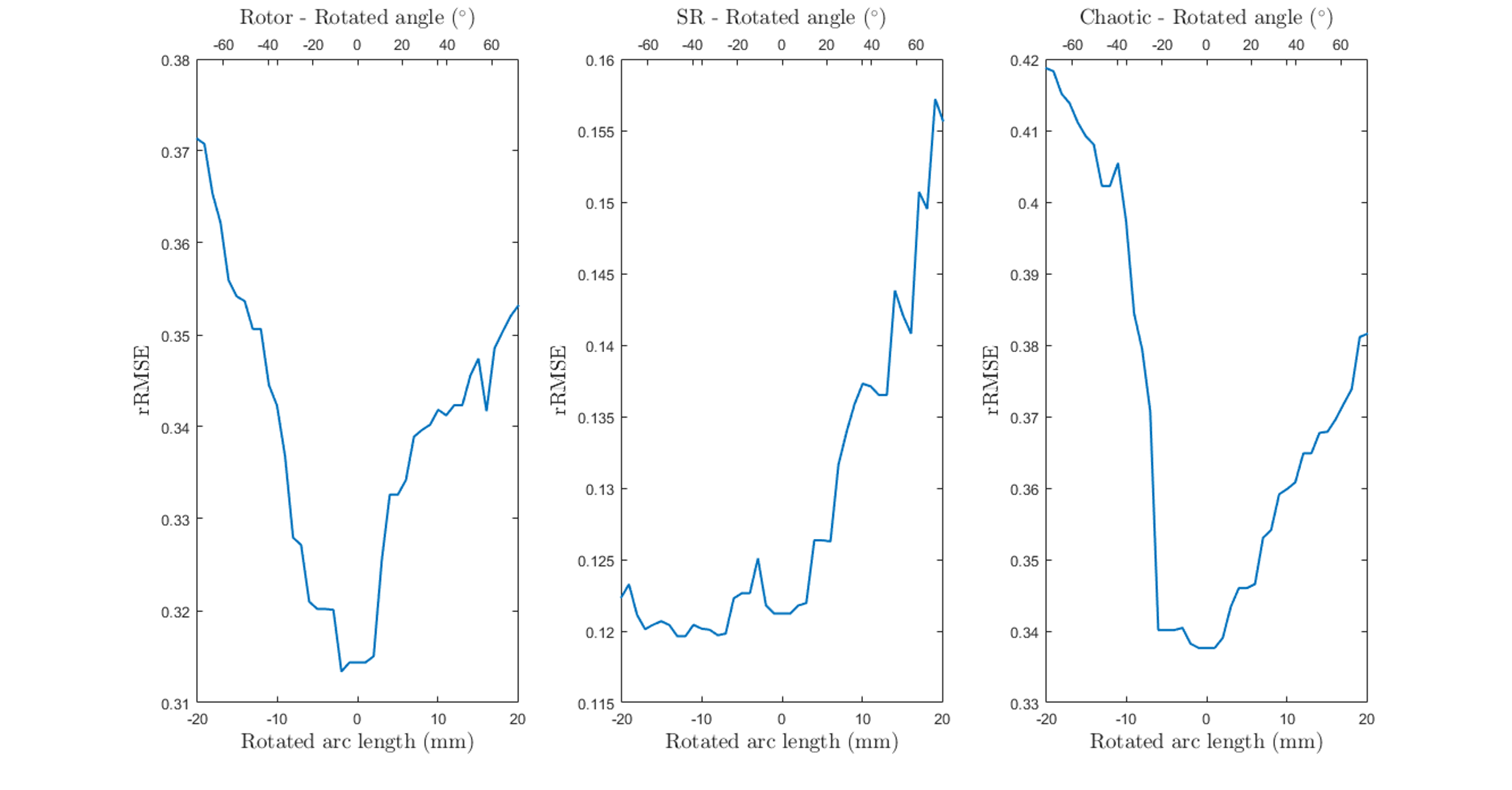

Supplement: Supplementary Figure 2 — The figure shows the rRMSE when the electrodes in a catheter branch are rotated an angle θ from the predefined interpolation position. The top x-axis covers the [−θ, θ] angle range for θ=2π5 radians (or 72°). The bottom x-axis is in linear units and represents the length of the rotated arc of the 32 mm circumference containing the most distal electrode of the PentaRay catheter. The figure displays the three in silico cases: rotor, SR and chaotic wavefronts. The rRMSE error remains almost identical for a shift of ± 2 mm. [file Image2.TIF]
